# Supplementary material for: The Estimated Intake of S100B Relates to Microbiota Biodiversity in Different Diets
Source: Biomolecules. 2025 Jul 18;15(7):1047. doi: 10.3390/biom15071047 (PMC12292894; doi:10.3390/biom15071047)
Supplement: Supplementary file 1 [file biomolecules-15-01047-s001.zip › biomolecules-3697913-Figure S1.pdf]

biomolecules

MDPI

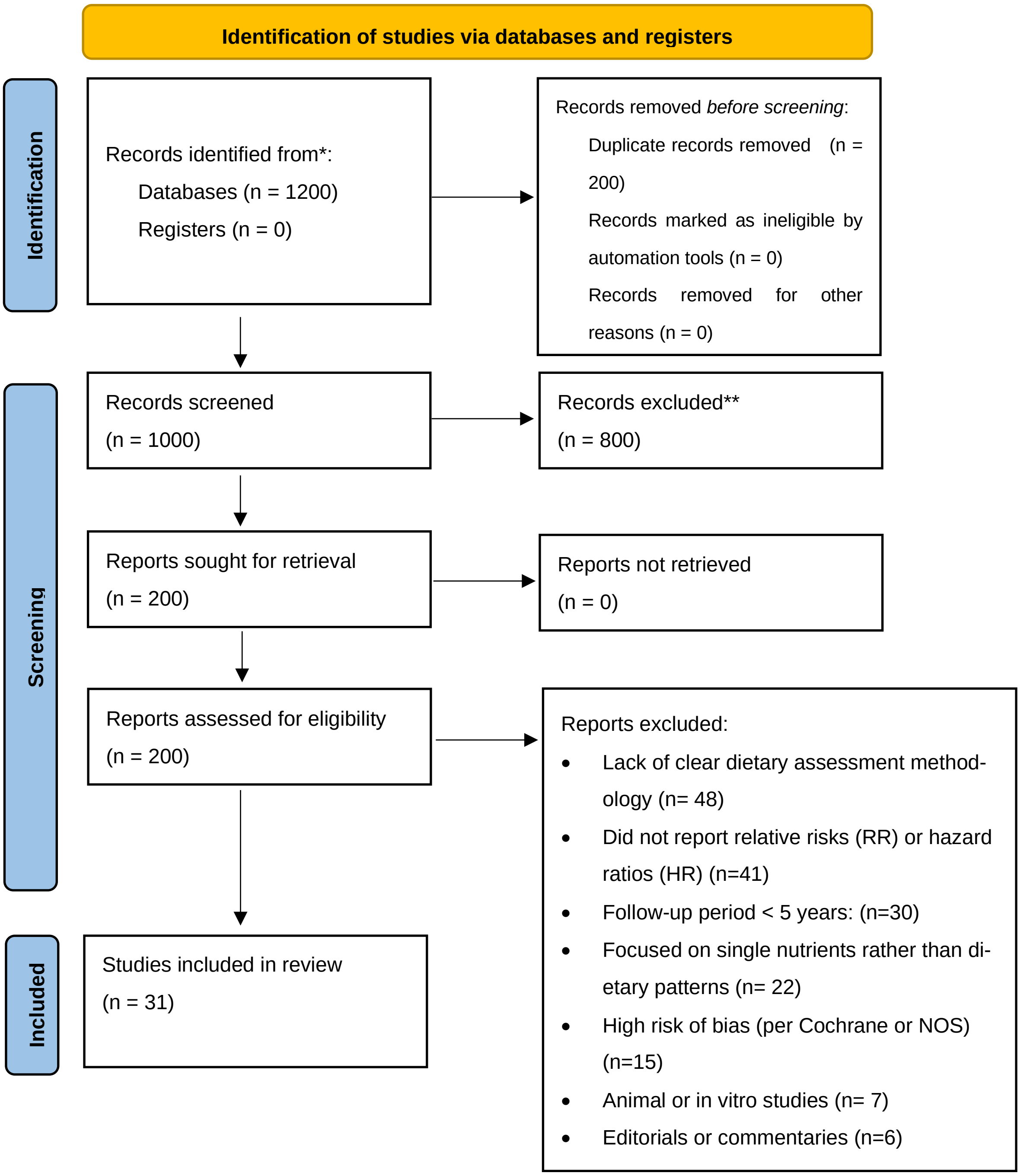

Figure S1. Selection process of the systematic literature review.

*Consider, if feasible to do so, reporting the number of records identified from each database or register searched (rather than the total number across all databases/registers).

**If automation tools were used, indicate how many records were excluded by a human and how many were excluded by automation tools.

This work is licensed under CC BY 4.0. To view a copy of this license, visit https://creativecommons.org/licenses/by/4.0/

References

1. Page, M. J.; McKenzie, J.E.; Bossuyt, P.M.; Boutron, I.; Hoffmann, T.C.; Mulrow, C.D.; Shamseer, L.; Tetzlaff, J.M.; Akl, E.A.; Brennan, S.E.; et al. The PRISMA 2020 statement: an updated guideline for reporting systematic reviews. *PLoS Med*. **2021** *18(3)*, e1003583. Https://doi.Org/10.1371/journal.pmed.1003583. PMID: 33780438; PMCID: PMC8007028.
