# Supplementary material for: The Estimated Intake of S100B Relates to Microbiota Biodiversity in Different Diets
Source: Biomolecules. 2025 Jul 18;15(7):1047. doi: 10.3390/biom15071047 (PMC12292894; doi:10.3390/biom15071047)
Supplement: Supplementary file 1 [file biomolecules-15-01047-s001.zip › biomolecules-3697913-Figure S2.pdf]

biomolecules

MDPI

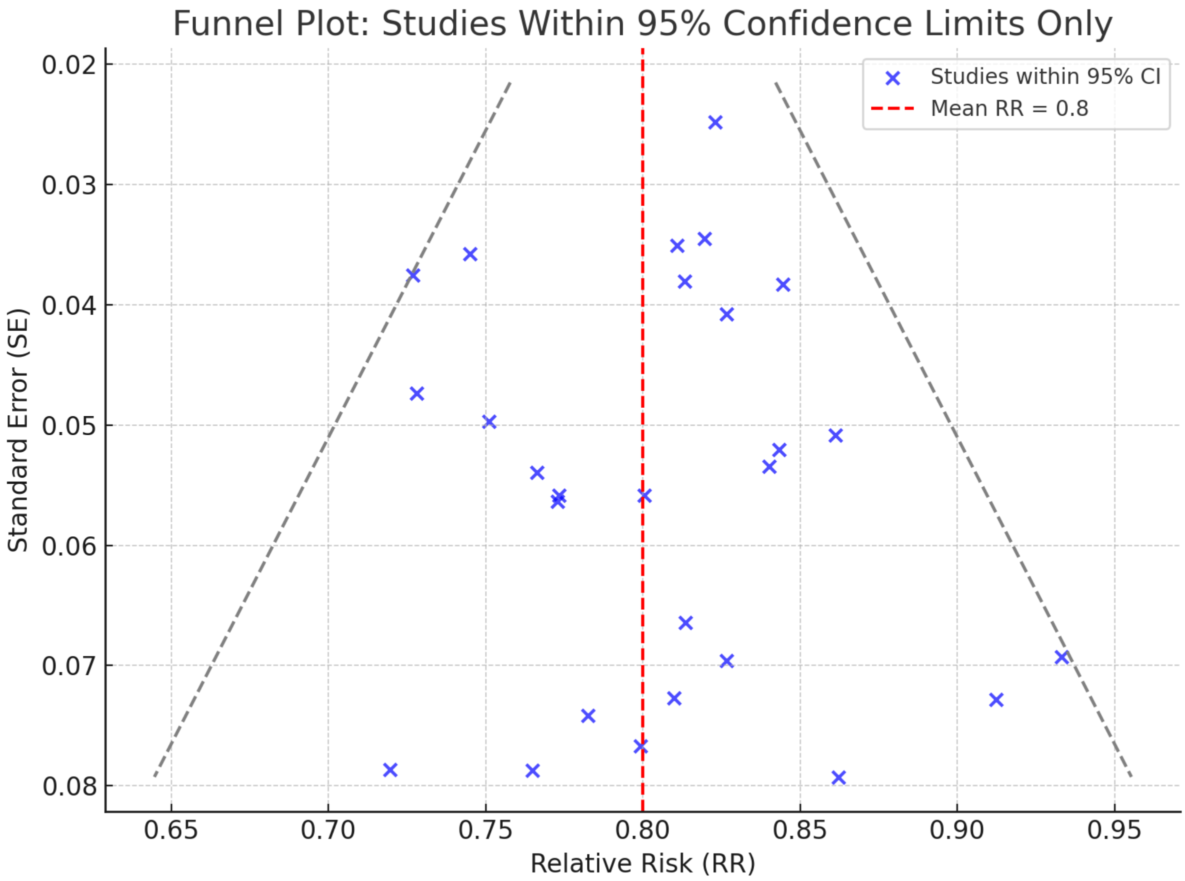

Figure S2. Funnel plot of the studies included in the meta-analysis evaluating the association between dietary patterns and chronic disease risk. Each dot represents an individual study, with the horizontal axis showing the relative risk (RR) and the vertical axis showing the standard error (SE). The red dashed line indicates the mean effect size. The gray dashed lines represent the pseudo 95% confidence limits. The symmetrical distribution of the studies suggests a low risk of publication bias, which is further supported by Egger's test (p = 0.032).
