# Supplementary material for: The Estimated Intake of S100B Relates to Microbiota Biodiversity in Different Diets
Source: Biomolecules. 2025 Jul 18;15(7):1047. doi: 10.3390/biom15071047 (PMC12292894; doi:10.3390/biom15071047)
Supplement: Supplementary file 1 [file biomolecules-15-01047-s001.zip › biomolecules-3697913-Table S1.pdf]

biomolecules

MDPI

Table S1. Characteristics of the 31 studies included in the meta-analysis, evaluating the association between dietary patterns and the risk of chronic diseases.

Author(s)	Year	Country	Sample Size
Li et al.	2018	USA	>800,000
Schwingshackl & Hoffmann	2015	Germany	N/A
Reedy et al.	2014	USA	N/A
Heidemann et al.	2008	USA	93,676
Shu et al.	2024	China	N/A
Tognon et al.	2012	Sweden	N/A
Liese et al.	2015	USA	N/A
Myint et al.	2009	UK	22,914
Shin et al.	2023	Korea	N/A
Castello et al.	2014	Spain	1017
Esposito et al.	2010	Italy	136,846
Kurotani et al.	2013	Japan	N/A
Kaluza et al.	2014	Sweden	41,541
Chan et al.	2015	Hong Kong	N/A
Kesse-Guyot et al.	2013	France	N/A
Hu et al.	2000	USA	51,529
Trichopoulou et al.	2003	Greece	22,043
Fung et al.	2001	USA	68,782
Knoops et al.	2004	Europe	2339
Esposito et al.	2004	Italy	180
Tucker et al.	2005	USA	894
Nöthlings et al.	2008	Europe	10,449
Mente et al.	2009	Global	N/A
Estruch et al.	2013	Spain	7447
Tognon et al.	2014	Denmark	57,053
Kokkinos et al.	2005	Greece	1212
Panagiotakos et al.	2008	Greece	3042
Panagiotakos et al.	2009	Greece	848
Kastorini et al.	2011	Greece	N/A
Panagiotakos et al.	2007	Greece	1514
Panagiotakos et al.	2005	Greece	3042
