# Supplementary material for: The Estimated Intake of S100B Relates to Microbiota Biodiversity in Different Diets
Source: Biomolecules. 2025 Jul 18;15(7):1047. doi: 10.3390/biom15071047 (PMC12292894; doi:10.3390/biom15071047)
Supplement: Supplementary file 1 [file biomolecules-15-01047-s001.zip › biomolecules-3697913-Table S2.pdf]

Table S2. Estimation of S100B mean values in different diets.

DIET	MILK	CHEESE	FRUITS & VEGE-TABLES	TOTAL S100B	Mean value
Okinawan diet	0.0075 - 45 μg/kg	0.00085- 0.05 μg/kg	0.012- 58 μg/kg	~ 0.02035- 103.05 μg/kg	**~ 51.53 ± 0.46 μg/kg**
Mediterranean diet	0.0075 – 45 μg/kg	0.00085- 0.05 μg/kg	0.0072- 115 μg/kg	~ 0.01555 – 160. 0 μg/kg	**~ 80.03 ± 0.39 μg/kg**
Nordic diet	0.0075 - 45μg/kg	0.00085- 0.05 μg/kg	0.012 – 108 μg/kg	~ 0.02035 – 153.05 μg/kg	**~ 76.53 ± 0.32 μg/kg**
West African diet	0.015 – 70 μg/kg	0.00085- 0.05 μg/kg	0.015-81 μg/kg	~ 0.03085 – 151.05 μg/kg	**~ 75.54 ± 0.32 μg/kg**
French Diet	0.0075 – 45 μg/kg	0.00085- 0.05 μg/kg	0.018- 85 μg/kg	~ 0.02635 – 126. 05 μg/kg	**~ 65.03 ± 0.18 μg/kg**
Korean Diet	0.0075 – 45 μg/kg	0.00085- 0.05 μg/kg	0.00612 – 53.4 μg/kg	~ 0.01447 – 98.45 μg/kg	**~ 49.23 ± 0.02 μg/kg**
Chinese Diet	0.0075 – 45 μg/kg	0.00085- 0.05 μg/kg	0.010 – 63 μg/kg	~ 0.01835 – 108.05 μg/kg	**~ 54.03 ± 0.16 μg/kg**
Thai Diet	0.0075 - 45 μg/kg	0.00085- 0.05 μg/kg	0.012 – 62.5μg/kg	~ 0.02035 – 112.05 μg/kg	**~ 53.03 ± 0.16 μg/kg**
Italian Diet	0.0075- 70 μg/kg	0.00085- 0.05 μg/kg	0.01212 – 120 μg/kg	~ 0.1295– 190.45 μg/kg	**~ 95.08 ± 0.23 μg/kg**
Indian Diet	0.0075 - 45 μg/kg	0.00085- 0.05 μg/kg	0.018- 81 μg/kg	~ 0.02635 – 126. 05 μg/kg	**~ 63.03 ± 0.37 μg/kg**
Pakistani & Bang-ladeshi Diet	0.0075 - 45 μg/kg	0.00085- 0.05 μg/kg	0.018- 81 μg/kg	~ 0.02635 – 126. 05 μg/kg	**~ 63.03 ± 0.37 μg/kg**
Middle East Diet	0.0075- 70 μg/kg	0.00085- 0.15 μg/kg	0.012- 50μg/kg	~ 0.02035 - 120.15 μg/kg	**~ 60.08 ± 0.22 μg/kg**
Western diet (American-style diet)	0.0075 – 45 μg/L	0.004-0.048 μg/kg	0.024 – 54 μg/kg	0.0355 – 99.048 μg/kg	**~ 49.54 ±0.43 μg/kg**

Table S2. Summary of reference values for S100B Calculations (from Michetti & Spica, 2025; Ghaffar et al ,2025).

**Farm animal milks**	0.03-180 μg/L
**Cheese-Diary**	0.01-0.4 μg/Kg
**Fruit-Vegetables**	0.04-180 μg/Kg

Reference:

1. Michetti, F., Romano Spica, V. The "Jekyll Side" of the S100B Protein: Its Trophic Action in the Diet. *Nutr.* **2025**, 17(5), 881. https://doi.org/10.3390/nu17050881.
2. Ghaffar, T.; Volpini, V.; Platania, S.; Glogowski, P.A.; Gianfranceschi, G.; Vassioukovitch, O.; Valeriani, F.; Michetti, F.; Romano Spica, V. A novel role for S100B in diet and gut-microbiota regulation. In Proceedings of the Gut Microbiota for Health World Summit 2025, Washington, DC, USA, 15–16 March 2025; [(accessed on 16 January 2024)]. Available online: https://agau.gastro.org/cw/course-details?entryId=17324988#nav-home.
