# Supplementary material for: The Estimated Intake of S100B Relates to Microbiota Biodiversity in Different Diets
Source: Biomolecules. 2025 Jul 18;15(7):1047. doi: 10.3390/biom15071047 (PMC12292894; doi:10.3390/biom15071047)
Supplement: Supplementary file 1 [file biomolecules-15-01047-s001.zip › biomolecules-3697913-Table S3.pdf]

biomolecules

MDPI

Table S3. Mini-review of data for estimation of Shannon index in different diets.

Type of Diet	Number of Papers	References	Shannon Index	Shannon Index (Mean value)
**Okinawan Diet**	2	1. Manoharan, L.; Roth, B.; Bang, C.; Stenlund, H.; Ohlsson, B. An Okinawan-Based Nordic Diet Leads to Profound Effects on Gut Microbiota and Plasma Metabolites Linked to Glucose and Lipid Metabolism. *Nutrients* 2023, *15*, 3273. https://doi.org/10.3390/nu15143273	2.60	2.70
		1. Huang, F.; Nilholm, C.; Roth, B.; Linninge, C.; Höglund, P.; Nyman, M., & Ohlsson, B. Anthropometric and metabolic improvements in human type 2 diabetes after introduction of an Okinawan-based Nordic diet are not associated with changes in microbial diversity or SCFA concentrations. *Int. J. Food Sci. Nutr.* **2018**, *69*(6), 729–740. https://doi.org/10.1080/09637486.2017.1408059.	2.80	
**Mediterrane An Diet**	6	1. Bourdeau-Julien, I.; Castonguay-Paradis, S.; Rochefort, G.; Perron, J.; Lamarche, B.; Flamand, N.; Di Marzo, V.; Veilleux, A.; Raymond, F. The diet rapidly and differentially affects the gut microbiota and host lipid mediators in a healthy population. *Microbiome* **2023**,*11*, 26. https://doi.org/10.1186/s40168-023-01469-2	3.40	3.00
		2. Gallè, F.; Valeriani, F.; Cattaruzza, M.S.; Gianfranceschi, G.; Liguori, R.; Antinozzi, M.; Mederer, B.; Liguori, G.; Romano Spica, V. Mediterranean Diet. Physical Activity and Gut Microbiome Composition: A Cross-Sectional Study among Healthy Young Italian Adults. *Nutrients* **2020**, *12(7)*, 2164. https://doi.org/10.3390/nu12072164. PMID: 32708278; PMCID: PMC7401267.	2.70	
		3. Garcia-Mantrana, I.; Selma-Royo, M.; Alcantara, C.; & Collado, M.C. . Shifts on gut microbiota associated to mediterranean diet adherence and specific dietary intakes on general adult population. *Front. Microbiol.* **2018**, *9*, 890. https://doi.org/10.3389/fmicb.2018.00890.	2.90	
		4. Wang, D.D.; Nguyen, L.H.; Li, Y.; Yan, Y.; Ma, W.; Rinott, E.; Ivey, K.L.; Shai, I.; Willett, W.C.; Hu, F.B.; Rimm, E.B.; Stampfer, M.J.; Chan, A.T.; Huttenhower, C. The gut microbiome modulates the protective association between a Mediterranean diet and cardiometabolic disease risk. *Nat. Med.* **2021**, *27(2)*, 333-343. https://doi.org/10.1038/s41591-020-01223-3.	2.90	
		5. Khavandegar, A.; Heidarzadeh, A.; Angoorani, P.; Hasani-Ranjbar, S.; Ejtahed, H.S.; Larijani, B.; & Qorbani, M. Adherence to the Mediterranean diet can beneficially affect the gut microbiota composition: A systematic review. *BMC Med. Genom.* **2024**, *17*(1), 91. https://doi.org/10.1186/s12920-024-01861-3.	3.10	
		6. Maldonado-Contreras, A.; Noel, S.E.; Ward, D.V.; Velez, M.; & Mangano, K.M. Associations between diet, the gut microbiome, and short-chain fatty acid production among older Caribbean	3.00	

		Latino adults. *J. Acad. Nutr. Diet.* **2020**, *120(12)*, 2047-2060. https://doi.org/10.1016/j.jand.2020.04.018.		
**Nordic Diet**	2	1. Roager, H.M.; Licht, T.R., Poulsen, S.K.; Larsen TM.; Bahl, M.I. Microbial enterotypes, inferred by the prevotella-to-bacteroides ratio, remained stable during a 6-month randomized controlled diet intervention with the new nordic diet. *Appl Env. Microbiol.* **2014** , 80(3), 1142–1149. https://doi.org/10.1128/AEM.03549-13	2.39	2.44
		2. Koponen, K.K.; Salosensaari, A.; Ruuskanen, M.O.; Havulinna, A.S.; Männistö, S.; Jousilahti, P.; Palmu, J.; Salido, R.; Sanders, K.; Brennan, C. et al. Associations of healthy food choices with gut microbiota profiles. *Am J Clin Nutr.* **2021**, *114*(2), 605-616. https://doi.org/10.1093/ajcn/nqab077	2.50	
**West African Diet**	5	1. Tang, M.; Frank, D.N., Tshefu, A.; Lokangaka, A., Goudar, S.S.; Dhaded, S.M.; Somannavar, M.S.; Hendricks, A.E.; Ir, D.; Robertson, C.E.; Kemp, J.F.; Lander, R.L.; Westcott, J.E.; Hambidge, K.M.; Krebs, N.F. Different Gut Microbial Profiles in Sub-Saharan African and South Asian Women of Childbearing Age Are Primarily Associated With Dietary Intakes. *Front. Microbiol.* **2019**, *10*, 1848. https://doi.org/10.3389/fmicb.2019.01848.	2.90	2.9
		2. Morton, E.R.; Lynch, J., Froment, A.; Lafosse, S., Heyer, E.; Przeworski, M.; Blekhman, R.; Ségurel, L. Variation in Rural African Gut Microbiota Is Strongly Correlated with Colonization by Entamoeba and Subsistence. *PLoS Genet.* **2015**, *11(11)*, e1005658. https://doi.org/10.1371/journal.pgen.1005658.	2.75	
		3. De, Filippo.; C.; Cavalieri; D.; Di Paola, M.; Ramazzotti; M.; Poullet; J.B.; Massart; S.; Collini; S.; Pieraccini; G.; Lionetti, P. Impact of diet in shaping gut microbiota revealed by a comparative study in children from Europe and rural Africa. *Proc. Natl. Acad. Sci.* **2010**; *107*(33); 14691-14696. https://doi.org/10.1073/pnas.1005963107.	3.00	
		4. Isibor, P.O.; Akinduti, P.A.; Aworunse, O.S.; Oyewale, J.O.; Oshamika, O.; Ugboko, H.U.; Taiwo, O.S.; Ahuekwe, E.F.; Obafemi, Y.D.; Onibokun, E.A. et al. Significance of African Diets in Biotherapeutic Modulation of the Gut Microbiome. *Bioinform Biol Insights* **2021**, 15, 11779322211012697. https://doi.org/10.1177/11779322211012697. PMID: 33994782; PMCID: PMC8107938.	2.85	
		5. Maigoro, A.Y.; Muhammad, M.; Bello, B.; Useh, U.; Lee, S. Exploration of Gut Microbiome Research in Africa: A Scoping Review. *J Med Food*. **2023**, *26(9)*, 616-623. https://doi.org/10.1089/jmf.2023.K.0005. PMID: 37523293.	3.00	
**French Diet**	2	1. Yasir, M.; Angelakis, E.; Bibi, F.; Azhar, E.I.; Bachar, D.; Lagier, J.C.; Gaborit, B.; Hassan, A.M.; Jiman-Fatani, A.A.; Alshali, K.Z.,; Robert, C.; Dutour, A.; Raoult, D. Comparison of the gut microbiota of people in France and Saudi Arabia. *Nutr Diabetes*. **2015**; 5(4); e153. https://doi.org/10.1038/nutd.2015.3.	3.00	2.9
		2. Partula, V.; Mondot, S.; Torres, M.J.; Kesse-Guyot, E.; Deschasaux, M.; Assmann, K.; Latino-Martel, P.; Buscail, C.; Julia, C.; Galan, P. et al. Milieu Intérieur Consortium. Associations between usual diet and gut microbiota composition: Results from the	2.80	

		Milieu Intérieur cross-sectional study. *Am J Clin Nutr*. **2019**; *109(5)*; 1472-1483. https://doi.org/10.1093/ajcn/nqz029.		
**Korean Diet**	2	1. Noh, H.; Jang, H-H.; Kim, G.; Zouiouich, S.; Cho, S-Y.; Kim, H-J.; Kim, J.; Choe, J-S.; Gunter, M.J.; Ferrari, P. et al. Taxonomic Composition and Diversity of the Gut Microbiota in Relation to Habitual Dietary Intake in Korean Adults. *Nutrients*. **2021**; *13(2)*; 366. https://doi.org/10.3390/nu13020366	1.35	1.42
		2. Nam, Y.D.; Jung, M.J.; Roh, S.W.; Kim, M.S.; Bae, J.W. Comparative analysis of Korean human gut microbiota by barcoded pyrosequencing. *PLoS One*. **2011**; *6(7)*; e22109. https://doi.org/10.1371/journal.pone.0022109. PMID: 21829445; PMCID: PMC3146482	1.50	
**Chinese Diet**	6	1. Wang, G.; Lyu, Q.; Yang, T.; Cui, S.; Niu, K.; Gu, R.; Li, Y.; Li, J.; Xing,W.; Li, L. Association of intestinal microbiota markers and dietary pattern in Chinese patients with type 2 diabetes: The Henan rural cohort study. *Front. Public Health* **2022**; *10*; 1046333. https://doi.org/10.3389/fpubh.2022.1046333. PMID: 36466492; PMCID: PMC9709334	2.90	2.19
		2. Ren, Y.; Wu, J.; Wang, Y.; Zhang, L.; Ren, J.; Zhang, Z.; Chen ,B.; Zhang, K.; Zhu, B.; Liu, W.; Li, S.; Li, X. Lifestyle patterns influence the composition of the gut microbiome in a healthy Chinese population. *Sci Rep*. **2023**; *13(1)*; 14425. https://doi.org/10.1038/s41598-023-41532-4. PMID: 37660184; PMCID: PMC10475076.	2.20	
		3. Jain; A.; Li, X.H..; Chen, W.N. Similarities and differences in gut microbiome composition correlate with dietary patterns of Indian and Chinese adults. *AMB Express* **2018**; *8(1)*; 104. https://doi.org/10.1186/s13568-018-0632-1. PMID: 29936607; PMCID: PMC6015586.	2.00	
		4. Jiang, X.; Wang, X.; Zhang, M.; Yu, L.; He, J.; Wu, S.; Yan, J.; Zheng, Y.; Zhou, Y.; Chen, Y. Associations between specific dietary patterns; gut microbiome composition; and incident subthreshold depression in Chinese young adults. *J Adv Res*. **2024**; *65*;183-195. https://doi.org/10.1016/j.jare.2024.05.030. PMID: 38879123; PMCID: PMC11518947.	2.10	
		5. Zhang, Y.; Chen, H.; Lu, M.; Cai, J.; Lu, B.; Luo, C.; Dai, M. Habitual Diet Pattern Associations with Gut Microbiome Diversity and Composition: Results from a Chinese Adult Cohort. *Nutrients* **2022**; *14(13)*; 2639. https://doi.org/10.3390/nu14132639. PMID: 35807820; PMCID: PMC9268000.	2.00	
		6. Zhang, S.; Ning, R.; Zeng, B.; Deng, F.; Kong, F.; Guo, W.; Zhao, J.; Li, Y. Gut Microbiota Composition and Metabolic Potential of Long-Living People in China. *Front Aging Neurosci* **2022**; *14*; 820108. https://doi.org/10.3389/fnagi.2022.820108. PMID: 35875797; PMCID: PMC9300991.	1.90	
**Thai Diet**	2	1. Sinsuebchuea, J.; Paenkaew, P.; Wutthiin, M.; Nantanaranon, T.; Laeman, K.; Kittichotirat, W.; Wattanachaisaereekul, S.; Dulsawat, S.; Nopharatana, M.; Vorapreeda, N. et al.Characterization of the Gut Microbiota in Urban Thai Individuals Reveals Enterotype-Specific Signature. *Microorg*. **2023**; *11(1)*; 136. https://doi.org/10.3390/microorganisms11010136. PMID: 36677429; PMCID: PMC9866083.	2.00	1.90

		2. Gruneck, L.; Kullawong, N.; Kespechara,; K.; Popluechai, S. Gut microbiota of obese and diabetic Thai subjects and interplay with dietary habits and blood profiles. *PeerJ.* **2020**; *8*; e9622. https://doi.org/10.7717/peerj.9622. PMID: 32832269; PMCID: PMC7409811.	1.80	
**Italian Diet**	6	1. Tagliamonte, S.; Puhlmann, M.L.; De Filippis, F.; Guerville M.; Ercolini, D.; Vitaglione, P. Relationships between diet and gut microbiome in an Italian and Dutch cohort: Does the dietary protein to fiber ratio play a role? *Eur J Nutr.* **2024**; *63(3)*; 741-750. https://doi.org/10.1007/s00394-023-03308-4. Epub 2023 Dec 27. PMID: 38151533; PMCID: PMC10948488	3.20	3.2
		2. Sisti, D.; Pazienza, V.; Piccini, F.; Citterio, B.; Baffone, W.; Donati Zeppa, S.; Biavasco, F.; Prospero, E.; De Luca, A.; Artico, M. et al. A proposal for the reference intervals of the Italian microbiota "scaffold" in healthy adults. *Sci Rep.* **2022**; *12(1)*; 3952. https://doi.org/10.1038/s41598-022-08000-x. PMID: 35273317; PMCID: PMC8913673.	3.30	
		3. Tavella, T.; Rampelli, S.; Guidarelli, G.; Bazzocchi, A.; Gasperini, C.; Pujos-Guillot, E.; Comte, B.; Barone, M.; Biagi, E.; Candela, M. et al. Elevated gut microbiome abundance of Christensenellaceae; Porphyromonadaceae and Rikenellaceae is associated with reduced visceral adipose tissue and healthier metabolic profile in Italian elderly. *Gut Microbes* **2021**; *13(1)*; 1-19. https://doi.org/10.1080/19490976.2021.1880221. PMID: 33557667; PMCID: PMC7889099.	3.00	
		4. Cerroni, R.; Pietrucci, D.; Teofani, A.; Chillemi, G.; Liguori, C.; Pierantozzi, M.; Unida, V.; Selmani, S.; Mercuri, N.B.; Stefani, A. Not just a Snapshot: An Italian Longitudinal Evaluation of Stability of Gut Microbiota Findings in Parkinson's Disease. *Brain Sciences* **2022**; *12(6)*; 739. https://doi.org/10.3390/brainsci12060739	3.20	
		5. Prinelli, F.; Jesuthasan, N.; Severgnini, M.; Musicco, M.; Adorni, F.; Correa, Leite, M.L.; Crespi; C.; Bernini; S. Exploring the relationship between Nutrition; gUT microbiota; and BRain AgINg in community-dwelling seniors: The Italian NutBrain population-based cohort study protocol. *BMC Geriatr.* **2020**; *20(1)*; 253. https://doi.org/10.1186/s12877-020-01652-2. PMID: 32703186; PMCID: PMC7376643.	3.20	
		6. Barone, M.; Turroni, S.; Rampelli, S.; Soverini, M.; D'Amico, F.; Biagi, E.; Brigidi, P.; Troiani;, E.; Candela, M. Gut microbiome response to a modern Paleolithic diet in a Western lifestyle context. *PLoS One* **2019**; *14(8);* e0220619. https://doi.org/10.1371/journal.pone.0220619.	3.30	
**Indian Diet**	2	1. Jain, A.; Li, XH.; Chen, W.N.Similarities and differences in gut microbiome composition correlate with dietary patterns of Indian and Chinese adults. *AMB Express* **2018**; *8(1)*; 104. https://doi.org/10.1186/s13568-018-0632-1. PMID: 29936607; PMCID: PMC6015586.	2.40	2.2
		2. Joshi, D.D.; Deb, L.; Kaul, K.; Somkuwar, B.G.; Rana, V.S.; Singh, R. Relevance of Indian Traditional Herbal Brews for Gut Microbiota Balance. *Indian J Microbiol.* **2024**; *64(4)*;1425-1444. https://doi.org/10.1007/s12088-024-01251-4. PMID: 39678955; PMCID: PMC11645388.	2.00	

**Pakistani & Bangladeshi Diet**	5	1. Ali, I.; Liu, K.; Long, D.; Faisal, S.; Hilal, M.G.; Ali, I.; Huang, X.; Long, R. Ramadan Fasting Leads to Shifts in Human Gut Microbiota Structured by Dietary Composition. *Front. Microbiol* **2021**; *12*; 642999. https://doi.org/10.3389/fmicb.2021.642999. PMID: 33679680; PMCID: PMC7930080	2.90	2.3
		2. Ahmad, A.; Yang, W.; Chen, G.; Shafiq, M.; Javed, S.; Ali Zaidi, S.S.; Shahid, R.; Liu, C.; Bokhari, H. Analysis of gut microbiota of obese individuals with type 2 diabetes and healthy individuals. *PloS one* **2019**; *14(12)*; e0226372. https://doi.org/10.1371/journal.pone.0226372.	2.10	
		3. Gul, F.; Herrema, H.; Davids, M.; Keating, C.; Nasir, A.; Ijaz; U.Z.; & Javed, S. Gut microbial ecology and exposome of a healthy Pakistani cohort. *Gut Pathogens* **2024**; *16(1)*; 5. https://doi.org/10.1186/s13099-024-00596-x.	2.30	
		4. Batool, M.; Ali, S.B.; Jaan, A.; Khalid, K.; Ali, S.A.; Kamal, K.; Nasir, A. Initial sequencing and characterization of gastrointestinal and oral microbiota in urban pakistani adults reveals abnormally high levels of potentially starch metabolizing bacteria in the general population. *BiorXiv*; **2018**; 419598. https://doi.org/10.1101/419598	2.00	
		5. Saleem, A.; Ikram, A.; Dikareva, E.; Lahtinen, E.; Matharu, D.; Pajari, A.M; de Vos, W.M.; Hasan, F.; Salonen, A.; Jian, C. Unique Pakistani gut microbiota highlights population-specific microbiota signatures of type 2 diabetes mellitus. *Gut Microbes* **2022**; *14(1)*; 2142009. https://doi.org/10.1080/19490976.2022.2142009.	1.90	
**Western Diet (Standard American Diet)**	3	1. García-Montero, C.; Fraile-Martínez, O.; Gómez-Lahoz, A.M.; Pekarek, L.; Castellanos, A.J.; Noguerales-Fraguas, F.; Coca, S.; Guijarro, L.G.; García-Honduvilla, N.; Asúnsolo, A. et al. Nutritional Components in Western Diet Versus Mediterranean Diet at the Gut Microbiota-Immune System Interplay. Implications for Health and Disease. *Nutrients* **2021**; *13(2)*; 699. https://doi.org/10.3390/nu13020699	1.00	1.0
		2. Ross, F.C.; Patangia, D.; Grimaud, G.; Lavelle, A.; Dempsey, E.M.; Ross, R.P.; Stanton, C. The interplay between diet and the gut microbiome: Implications for health and disease. *Nat. Rev. Microbiol. 2024*; *22*(11); 671-686. https://doi.org/10.1038/s41579-024-01068-4.	1.10	
		3. Frioux, C.; Ansorge, R.; Özkurt, E.; Ghassemi Nedjad, C.; Fritscher, J.; Quince, C.; Waszak, S.M.; Hildebrand; F. Enterosignatures define common bacterial guilds in the human gut microbiome. *Cell Host Microbe* **2023**; *31(7)*; 1111-1125.e6. https://doi.org/10.1016/j.chom.2023.05.024.	1.20	
**Middle East Diet**	2	1. Aljazairy, E.A.; Al-Musharaf, S.; Abudawood, M.; Almaarik, B.; Hussain, S.D.; Alnaami, A.M.; Sabico, S.; Al-Daghri, N.M.; Clerici, M.; Aljuraiban, G.S. Influence of Adiposity on the Gut Microbiota Composition of Arab Women: A Case-Control Study. *Biology (Basel)* **2022**; *11(11)*; 1586. https://doi.org/10.3390/biology11111586.	1.90	1.5
		2. Syromyatnikov, M.; Nesterova, E.; Gladkikh, M.; Smirnova, Y.; Gryaznova, M.; & Popov; V. Characteristics of the gut bacterial composition in people of different nationalities and religions. *Microorg.* **2022**; *10(9)*; 1866. https://doi.org/10.3390/microorganisms10091866.	1.20	
