# Supplementary material for: The Estimated Intake of S100B Relates to Microbiota Biodiversity in Different Diets
Source: Biomolecules. 2025 Jul 18;15(7):1047. doi: 10.3390/biom15071047 (PMC12292894; doi:10.3390/biom15071047)
Supplement: Supplementary file 1 [file biomolecules-15-01047-s001.zip › biomolecules-3697913-Table S4.pdf]

biomolecules

MDPI

Table S4. Estimation of S100B values in different foods.

Edible food	S100B concentration
Human milk	10.41 ± 4.2 microg/L
Cow milk	3.13 ±0.56 microg/L
Donkey milk	1.17 ± 0.26 microg/L
Sheep milk	0.25 ±0.11 microg/L
Goat milk	0.26 ± 0.11 microg/L
Sage (fresh)	180 microg /mg
Açai (powder)	17.15 microg /mg
Kombucha (powder)	8.99 microg /mg
Baobab (powder)	7.08 microg /mg
Laurel (fresh)	4.37 microg /mg
Durian (fresh)	3.4 microg /mg
Jack fruit (lyophilized)	0.32 microg /mg
Durian (powder)	0.012 microg /mg
Ancient apples	324.76 pg/ml
Cow Ricotta	264.39 pg/ml
Sheep Ricotta	265.80 pg/ml
